# Screening of Selected Stingless Bee Honey Varieties for ACE2-Spike Protein-Binding Inhibition Activity: A Potential Preventive Medicine Against SARS-Cov-2 Infection

**DOI:** 10.21315/mjms2022.29.5.15

**Published:** 2022-10-28

**Authors:** Enos Tangke Arung, Rico Ramadhan, Liidza Diana Mandzilkh, Prasetia Aktavinaldy Santoso, Masako Matsumoto, Maki Nagata, Irawan Wijaya Kusuma, Swandari Paramita, Sukemi, Yadi, Naomichi Takemoto, Yhiya Amen, Syafrizal, Kuniyoshi Shimizu, Nataniel Tandirogang

**Affiliations:** 1 Laboratory of Forest Product Chemistry, Faculty of Forestry, Mulawarman University, Samarinda, Indonesia; 2 Research Center for Drugs and Cosmetics from Tropical Rainforest Resources, Mulawarman University, Samarinda, Indonesia; 3 Department of Chemistry, Faculty of Science and Technology, Airlangga University, Surabaya, Indonesia; 4 Division of Exploration and Synthesis of Bioactive Compounds, Research Center for Bio-Molecule Engineering, Airlangga University, Surabaya, Indonesia; 5 Department of Agro-Environmental Sciences, Faculty of Agriculture, Kyushu University, Fukuoka, Japan; 6 Department of Community Medicine, Faculty of Medicine, Mulawarman University, Samarinda, Indonesia; 7 Chemical Education Program, Faculty of Teacher Training and Education, Mulawarman University, Samarinda, Indonesia; 8 Department of Microbiology, Faculty of Medicine, Mulawarman University, Samarinda, Indonesia; 9 Department of Pharmacognosy, Faculty of Pharmacy, Mansoura University, Mansoura, Egypt; 10 Department of Biology, Faculty of Mathematics and Science, Mulawarman University, Samarinda, Indonesia; 11 Institute for Asian and Oceanian Studies, Kyushu University, Fukuoka, Japan

**Keywords:** stingless bee, honey, phytochemical, ACE2-spike protein-binding, SARS-CoV-2

## Abstract

The broader objective of this study is to identify natural materials that might inhibit the severe acute respiratory syndrome coronavirus 2 (SARS-CoV-2) infection. We have focused on stingless bee honey, which has a unique taste that is both sweet and sour and sometimes bitter. We screened 12 samples of honey from 11 species of stingless bees using an angiotensin-converting enzyme 2 (ACE2)-spike protein-binding assay and phytochemical analysis. Ten of the samples showed inhibition above 50% in this assay system. Most of the honey contained tannins, alkaloids, flavonoids, triterpenoids, carotenoids and carbohydrates. Our findings in this in vitro study showed that honey from stingless bees may have a potent effect against SARS-CoV-2 infection by inhibiting the ACE2-spike protein-binding.

## Introduction

In December 2019, the severe acute respiratory syndrome coronavirus 2 (SARS-CoV-2) was detected in Wuhan, China. This disease spread rapidly, became an epidemic in China and has been found in 27 other countries (1). By January 2020, the Chinese Centre for Disease Control and Prevention had isolated this virus from a throat swab sample of a patient in Wuhan and it was subsequently named 2019-nCoV. It was renamed COVID-19 by the World Health Organization (2) and soon became a worldwide pandemic. Unfortunately, up to now, there are no registered specific therapeutics for this virus except for the available vaccines that are currently being challenged by several new variants. There is an urgent need to seek alternative approaches to preventing and controlling the replication and spread of this virus. The current management of the virus includes travel restrictions, patient isolation and supportive medical care (1, 3).

SARS-CoV-2 infects host cells by interacting with membrane-bound angiotensin-converting enzyme 2 (ACE2) in human tissues (4). Cell entry of SARS-CoV is mediated by the viral spike (S) protein as the receptor-binding domain (RBD). This RDB can trigger the endocytosis of susceptible cells. The S protein requires ACE2 molecules to attach at the cell surface to enable cell membrane fusion (5–7). Therefore, S protein-binding inhibition could provide a valuable strategy for preventing the membrane fusion of SARS-CoV-2 and its entry into human cells. Indeed, Zhang and Liu (3) reported that potential specific treatments for coronavirus include treatment with Corona viral protease inhibitors and ACE2-spike protein binding.

Stingless bee products include honey, pollen, wax and propolis/cerumen. Among these products, honey is the most valuable. It was associated with various health functions by ancient people and has been used as an income source for many generations (8). Stingless bee honey is different from that of *Apis mellifera* (honeybee). Several significant differences include higher water content, lower diastase activity and a different sugar spectrum (9). In our previous works on stingless bee products, we found that the honey of the *Wallacetrigona incisa* (formerly *Trigona incisa*) and *Tetragonula fuscobalteata* (formerly *Trigona fuscobalteata*) showed cytotoxicity effects against five human cancer cell lines, including the HepG2, SW620, ChaGo-I, KATO-III and BT474 cell lines (10). In the present study, we continue to investigate the medicinal properties of honey from 11 stingless bee species, including *Tetragonula biroi, Homotrigona fimbriata, Tetragonula sarawakensis, Tetragonula laeviceps, Tetragonula reepeni, Tetragonula fuscobalteata, Lepidotrigona terminata, Tetragonula testaceitarcis, Tetragonula iridipennis, Heterotrigona itama* and *Wallacetrigona incisa* to determine their potency as potential preventive treatments against COVID-19 infection through their ability to inhibit ACE2-spike protein-binding.

## Methods

### Chemicals

The spike RBD SARS-Cov-2: ACE2 inhibitor screening assay kit was purchased from the BPS Bioscience (San Diego, USA). Other chemicals used in this experiment were of the highest grade commercially available.

### Honey Collection and Preparation for Analysis

The stingless bee honey was collected from cities in Indonesia, including Balikpapan (*Tetragonula biroi*), Samarinda (*Homotrigona fimbriata*, *Tetragonula sarawakensis, Tetragonula laeviceps, Tetragonula reepeni, Tetragonula fuscobalteata*, *Lepidotrigona terminata*, *Tetragonula testaceitarsis*, *Tetragonula iridipennis* and *Heterotrigona itama*) and Palopo (*Wallacetrigona incisa*, two varieties of honey, one with a pineapple taste and the other with a bitter taste). The honey was collected from December 2019 through March 2020. The *Tetragonula biroi* honey was taken from the cultivation area in the middle of a palm oil plantation in Balikpapan. The honey from *Homotrigona fimbriata*, *Tetragonula sarawakensis, Tetragonula. laeviceps, Tetragonula reepeni, Tetragonula fuscobalteata*, *Tetragonula terminata*, *Tetragonula testaceitarsis*, *Tetragonula iridipennis* and *Heterotrigona itama* were collected from a cultivated area in a secondary forest in Samarinda and the *Wallacetrigona incisa* honeys with pineapple and bitter tastes were both from Palopo. All collected honey was stored in the refrigerator (4 °C). The honey was kept at room temperature for 30 min–60 min before the phytochemical analysis, diluted in water and then assayed with the ACE2-spike protein-binding assay.

### ACE2-Spike Protein-Binding Inhibition Assay

In this analysis, the honeys were diluted with water to 2%, 1%, 0.4% and 0.2% (v/v) concentrations. The ACE2-spike protein-binding inhibition assay was performed using the ACE2-spike protein-binding assay kit following the manufacturer’s instructions (5). In brief, purified ACE2 protein was attached to a nickel-coated 96-well plate. Then 10 μL of each sample was put into the wells, and spike protein was added to the plate and incubated with ACE2. Finally, horseradish peroxidase (HRP)-labeled antibody was added; then the sample was admixed with HRP substrate to produce chemiluminescence. The chemiluminescence was measured using a Wallac 1420 ARVO MX plate reader (Perkin Elmer Japan) at the Center for Advanced Instrumental and Educational Supports, Faculty of Agriculture, Kyushu University, Japan.

### Phytochemical Screening

Phytochemical tests were used to screen for the presence of tannins, alkaloids, flavonoids, terpenoids, steroids, carotenoids, coumarins and saponins. Carbohydrates were also tested qualitatively. The screening tests for these major phytochemicals and carbohydrates were carried out using standard qualitative procedures with some modifications (11, 12).

#### Detection of Tannins

One millilitre of honey was mixed with three drops of freshly prepared 1% lead acetate. The formation of yellow precipitates was considered a positive result for the presence of tannins (11).

#### Detection of Alkaloids

One millilitre of honey was mixed carefully with 2 mL of HCl in the test tube and 1 mL of Dragendorff reagent was added. The formation of a yellow precipitate indicated the presence of alkaloids in the honey (11).

#### Detection of Flavonoids

One millilitre of honey was treated with five drops of 1% sodium hydroxide solution. The formation of an intense yellow colour, which disappeared into a colourless solution with the addition of 1% HCl, indicated flavonoids in the honey (12).

#### Detection of Triterpenoids and Steroids

A mixture of 10 drops of acetic anhydride and two drops of concentrated sulfuric acid was added to 1 mL of honey diluted in acetone. The resultant mixture was shaken vigorously. A colour change to red or purple indicated the presence of triterpenoids, while a blue-green colour signified that steroids were present (11).

#### Detection of Carotenoids

One millilitre of honey was diluted with 5 mL of chloroform in a test tube and shaken vigorously and four drops of 85% sulfuric acid were then added. A blue colour on the surface of the mixtures indicated the presence of carotenoids (12).

#### Detection of Coumarins

One millilitre of honey was treated with four drops of sodium hydroxide and alcohol. A colour change to yellow indicated the presence of coumarins (12).

#### Detection of Saponins

One millilitre of honey was mixed with 2 mL of acetone and 3 mL hot water was added. The mixture solution was cooled and then shaken vigorously for 10 s. The formation of bubbles or persistent foam 1 cm–10 cm high for 10 min following the addition of one drop of HCl 2N and continuing froth, indicated the presence of saponins in the honey (12).

#### Detection of Carbohydrates

One millilitre of honey was dissolved in 1 mL of acetone in a test tube and then treated with a drop of Molisch’s reagent. The resultant mixture was shaken vigorously and treated with 1 mL of sulfuric acid concentration. Purple rings between the two layers of the mixture indicated the presence of carbohydrates (12).

## Results

**Figure 1:**
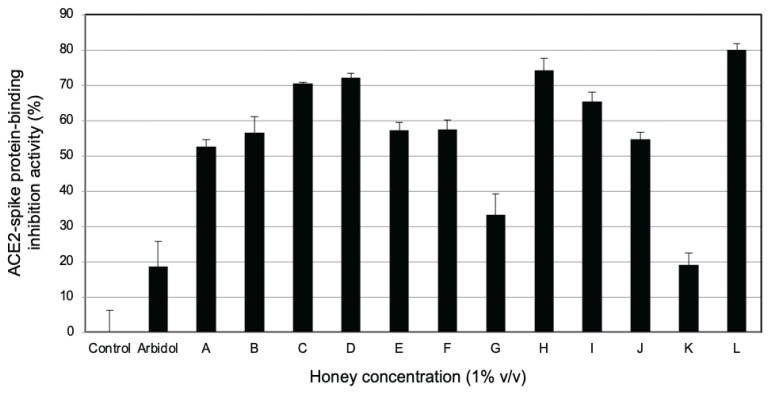
ACE2-spike protein-binding inhibition activity of stingless bee honey Notes: Control = arbidol, positive control (1 μg/mL); A = *Tetragonula biroi*; B = *Homotrigona fimbriata*; C = *Tetragonula sarawakensis*; D = *Tetragonula laeviceps*; E = *Tetragonula reepeni*; F = *Tetragonula fuscobalteata*; G = *Tetragonula terminata*; H = *Tetragonula testaceitarsis*; I = *Tetragonula iridipennis*; J = *Heterotrigona itama*; K = *Wallacetrigona incisa* (pineapple taste); L = *Wallacetrigona incisa* (bitter taste)

Figure 1 shows that the honey of *Wallacetrigona incisa* stingless bee (with a bitter taste) inhibited 80.0% of the binding of this protein. This rate of inhibition was followed by those of *Tetragonula testaceitarsis* (74.2%), *Tetragonula laeviceps* (72.2%), *Tetragonula sarawakensis* (70.5%), *Tetragonula iridipennis* (65.5%), *Tetragonula fuscobalteata* (57.6%), *Tetragonula reepeni* (57.4%), *Homotrigona fimbriata* (56.6%), *Heterotrigona itama* (54.8%), *Tetragonula biroi* (52.8%), *Lepidotrigona terminata* (33.4%) and *Wallacetrigona incisa* (pineapple taste) (19.1%).

**Figure 2:**
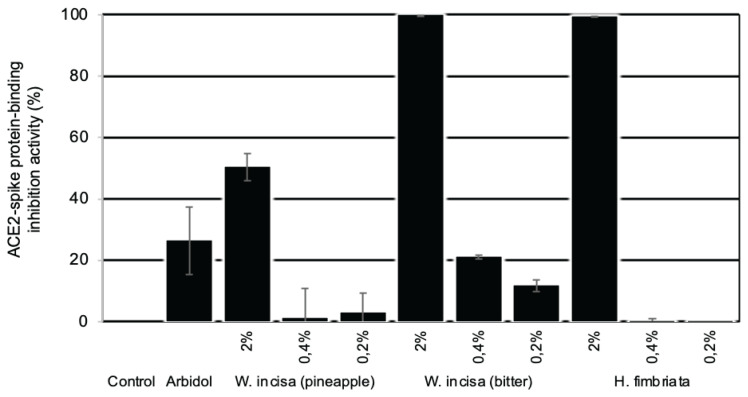
Dose-dependent test of selected stingless bee honey in ACE2-spike protein-binding inhibition activity Notes: Honey concentration (v/v); Arbidol = positive control (0.2 μg/mL); *W. incisa* = *Wallacetrigona incisa*; *H. fimbriata* = *Homotrigona fimbriata*

The rate for arbidol, the positive control, was 18.7% at 1 mg/mL. Arbidol has been used as an antiviral treatment and is known to block the trimerisation of the spike glycoprotein (3, 13). Figure 2 depicts the different inhibition rates at 2.0%, 0.4% and 0.2% concentrations. At the 2.0% concentration, *Wallacetrigona incisa* honey (bitter taste) showed the strongest inhibition of the binding (99.7%), followed by *Homotrigona fimbriata* (99.4%) and *Wallacetrigona incisa* (pineapple taste) (50.3%). At the concentrations of 0.4% and 0.2%, the inhibition provided by *Wallacetrigona incisa* (pineapple taste) and *Homotrigona fimbriata* honey decreased dramatically while that of *Wallacetrigona incisa* honey (bitter taste) decreased gradually.

By several assays, we determined the phytochemical contents qualitatively in 12 samples of honey. Table 1 shows that all honey samples had flavonoid and carbohydrate compounds while only *Tetragonula laeviceps* had steroid and saponin compounds.

## Discussion

Various health effects of honey-bee and stingless-bee honey have been previously demonstrated. These include antimicrobial, antioxidant and anti-inflammatory effects as well as other therapeutic effects for eye diseases, gastrointestinal diseases, neurology disorders, fertility disorders, diabetes, unhealthy cholesterol levels, cardiovascular diseases, wounds and cancer (14–16). These various health effects of stingless bee honey led us to investigate 12 selected honeys from 11 types of stingless bee using an ACE2-spike protein-binding assay as shown in Figure 1 at a concentration of 1% (v/v). We selected three honey samples with unique tastes and tested their dose-dependent inhibition activity based on these results. The samples chosen were Homotrigona fimbriata honey with an agathis or pine sap taste or aroma, Wallacetrigona incisa honey with a pineapple taste and Wallacetrigona incisa honey with a bitter taste.

The results of the honey’s ACE2-spike protein-binding inhibition assay directed us to investigate the phytochemicals in those honeys. As Table 1 shows, all honey samples contained flavonoid and carbohydrate compounds while only *Tetragonula laeviceps* contained steroid and saponin compounds. Cianciosi et al. (17) summarised that honey contains about 180 compounds, including water, sugars, free amino acids, proteins, enzymes, minerals, vitamins and phytochemicals. In addition, Al-Hatamleh et al. (18) reported that phenolic compounds, including phenolic acids and polyphenols (such as flavonoids), are mainly found in honey with diverse chemical structures. The most abundant phenolic acids are gallic acid, chlorogenic acid, syringic acid, vanillic acid, p-coumaric acid, p-hydroxybenzoic acid and caffeic acid, while the most abundant flavonoids are apigenin, quercetin, luteolin, chrysin, kaempferol, galangin, genistein, pinocembrin and pinobanksin. As shown in Figures 1 and 2, *Wallacetrigona incisa* honey (pineapple taste), which showed low inhibition of ACE2-spike protein-binding, and *Wallacetrigona incisa* honey (bitter taste), which showed high inhibition of ACE2-spike protein-binding, had different contents of carotenoid and coumarin compounds. The *Wallacetrigona incisa* honey with pineapple and bitter tastes came from different villages in the city of Palopo. Therefore, the high inhibition of ACE2-spike protein-binding provided by *Wallacetrigona incisa* honey (bitter taste) might be related to the coumarin rather than the carotenoid, which originates from a different nectar or vegetation source, geography and climate (18).

Phytochemicals of stingless bee honey are related to the plant sources: nectars, pollens, resins and oils. Nectar, the main substance from which honey is produced, contains different phytochemicals depending on the plant of origin. The resulting honey is stored in a pot as cerumen (a mixture of resin, bee salivary and beeswax) and the presence of phytochemicals in the cerumen may also affect the phytochemicals in potted honey (15). This phenomenon needs to be clarified with further analysis and requires more honey samples to obtain or determine the active compounds. The presence of phytochemicals may affect the ACE2-spike protein-binding, as mentioned by Zhang and Liu (3). They found that use as a coronavirus protease inhibitor required compounds such as cinanserin (alkaloid), flavonoid, diarylheptanoid and spike protein ACE2 blockers such as chloroquine (alkaloid), emodin (polyphenol), promazine (alkaloid) and nicotinamide (alkaloid).

Flavonoids and tannins were detected in almost all honey and these compounds possibly relate to the inhibition of spike protein-binding. Several flavonoids such as hesperidin (18), fisetin, quercetin, isorhamnetin, kaempferol, myricetin, herbacetin, morin, quercetin-*O*-pentoside, quercetin-*O*-rhamnoside, kaempferol-*O*-pentoside, quercetin-3-*O*-vicianoside, quercetin-7-*O*-galactoside, quercetin-3-*O*-rutinose, and quercetin-3-*O*-glucuronide-7-*O*-glucoside have shown ACE2-spike protein-binding inhibition comparable to that of known inhibitors such as chloroquine (an alkaloid) (19). Also, tannic acid has been shown to have antiviral effects against several viruses, including influenza A virus, papillomavirus, noroviruses, herpes simplex virus types 1 and 2 and HIV (20).

## Conclusion

In the current study, 10 samples of honey exhibited ACE2-spike protein-binding inhibition, which might be related to the phytochemical content in the honey. These findings indicate that honey from stingless bees could potentially be used to combat the SARS-CoV-2 pandemic. Further experiments are needed to clarify the active compounds and the mechanism by which they inhibit the spike protein-binding of this virus, as well as to examine the effects of ingestion of these substances on viral transmission.

## Figures and Tables

**Table 1:** Phytochemicals of selected stingless bee honey

No.	Honey	Phytochemicals								

		Tan	Alk	Fla	Tri	Ste	Car	Cou	Sap	Cbo
1	*Tetragonula biroi*	+	+	+	−	−	−	+	−	+
2	*Homotrigona fimbriata*	−	+	+	−	−	−	+	−	+
3	*Tetragonula sarawakensis*	+	+	+	−	−	−	+	−	+
4	*Tetragonula laeviceps*	+	−	+	−	+	−	−	+	+
5	*Tetragonula reepeni*	+	+	+	+	−	−	−	−	+
6	*Tetragonula fuscobalteata*	+	+	+	+	−	+	+	−	+
7	*Lepidotrigona terminata*	+	+	+	+	−	+	+	−	+
8	*Tetragonula testaceitarsis*	+	−	+	+	−	+	+	−	+
9	*Tetragonula iridipennis*	+	−	+	+	−	+	+	−	+
10	*Heterotrigona itama*	−	−	+	+	−	+	+	−	+
11	*Wallacetrigona incisa* (pineapple)	+	+	+	+	−	+	−	−	+
12	*Wallacetrigona incisa* (bitter)	+	+	+	+	−	−	+	−	+
